# Characterizing active transportation mechanisms for free fatty acids and antibiotics in *Synechocystis* sp. PCC 6803

**DOI:** 10.1186/s12896-019-0500-3

**Published:** 2019-01-10

**Authors:** Matthew P. A. Bellefleur, Soo-Young Wanda, Roy Curtiss

**Affiliations:** 1 School of Life Sciences, Arizona State University, 427 E. Tyler Mall, Tempe, AZ 85287 USA; 2 College of Veterinary Medicine, University of Florida, 2015 SW 16th Ave, Gainesville, FL 32608 USA

**Keywords:** Free fatty acids, Active transportation, IC_50_, Multidrug efflux

## Abstract

**Background:**

*Synechocystis* sp. PCC 6803 is a photosynthetic bacterium that has been genetically modified to produce industrially relevant chemicals, yet efflux mechanisms have not been well elucidated. These photosynthetic organisms live in environments that are often nutrient limited; therefore, the genome of these organisms encodes far fewer proteins used for efflux of chemicals when compared to members of the *Enterobacteriaceae* family. Understanding efflux mechanisms can lead to a greater efficiency of chemical production within the cyanobacterial cell.

**Results:**

Both *sll0180* and *slr2131* genes that encode the Sll0180 and Slr2131 proteins, respectively, were removed from *Synechocystis* sp. PCC 6803 and SD277, a high fatty acid-producing *Synechocystis*-based strain, to test the hypothesis that Sll0180 and Slr2131 contribute to the efflux of chemicals out of *Synechocystis* sp. PCC 6803 and SD277. The mutant *Synechocystis* sp. PCC 6803 and SD277 strains with either *sll0180* or *slr2131* removed from the chromosome had significantly decreased half maximal inhibitory concentrations to various antibiotics. The free fatty acid (FFA) concentration of the SD277 mutant strains increased intracellularly yet decreased extracellularly indicating that Sll0180 and Slr2131 have a role in FFA efflux. *E. coli* wild-type gene *acrA* (a homolog to *sll0180*) was added on a plasmid to the respective mutant strains lacking the *sll0180* gene. Similarly, the *E. coli* wild-type gene *acrB* (a homolog to *slr2131*) was added to the respective mutant strains lacking the *slr2131* gene. The tolerance to chloramphenicol of each mutant strain containing the wild-type *E. coli* gene was restored when compared to the parent stains. The extracellular FFA concentration of SD277 Δ*slr2131* with *E. coli acrB* increased significantly compared to both SD277 and SD277 Δ*slr2131*.

**Conclusions:**

Two proteins involved in the transportation of antibiotics and FFAs out of the *Synechocystis* sp. PCC 6803 cell were identified. In an effort to alleviate costs associated with mechanically or chemically separating the cells from the FFAs, the combination of genome editing of SD277 and the addition of exogenous transport gene increased extracellular concentrations of FFAs. This understanding of active transportation is critical to improving the production efficiency for all industrially relevant chemicals produced in *Synechocystis* sp. PCC 6803.

**Electronic supplementary material:**

The online version of this article (10.1186/s12896-019-0500-3) contains supplementary material, which is available to authorized users.

## Background

*Synechocystis* sp. PCC 6803 was isolated in 1968 from a freshwater lake [1] and was the first phototrophic organism whose whole genome was completely sequenced [2]. Prior to sequencing, *Synechocystis* sp. PCC 6803 was shown to be naturally transformable [3] and later, able uptake plasmid DNA through conjugation [4]. The combination of these findings led to the use of *Synechocystis* sp. PCC 6803 as a model organism for investigating a variety of fields of study including the process of photosynthesis and the use of cyanobacterial cells to produce biofuels. However, there remain gaps of knowledge as to the function of many proteins in the *Synechocystis* sp. PCC 6803 cell.

Genetically modified *Synechocystis* sp. PCC 6803 strains produce useful chemicals such as ethanol, acetone, free fatty acids (FFAs), and 3-hydroxybutyrate [5–8]. However, there is limited understanding as to how the chemicals are transported out of the cell. Much of the chemical transportation research throughout the last 15 years focused on the process by which chemicals are transported into *Synechocystis* sp. PCC 6803 cells. Some examples include the Fut series of proteins that are responsible for uptake of iron from the environment [9–11] and the Pst proteins that are responsible for phosphate uptake [12, 13]. While uptake of nutrients is paramount to understanding ecological survival of cyanobacteria with respect to algal blooms that create anoxic or toxin-filled waterways, understanding the native chemical efflux mechanisms is vital for using the cyanobacteria to produce industrially relevant chemicals. Once the industrially relevant chemicals are synthesized inside the cyanobacterial cell, the chemicals need to be expelled for two reasons: first, altered intracellular chemical concentrations can cause chemical or protein synthesis to cease or inhibit cell growth and second, the cells do not have to be lysed if the chemicals are in the supernatant, saving time and money. By promoting the secretion of the industrially relevant chemicals, the cyanobacteria can not only survive, they can also grow continuously if the chemical can be purified from the extracellular environment. In an effort to understand a mechanism of efflux of *Synechocystis* sp. PCC 6803, the function of a *Escherichia coli* TolC homolog, Slr1270, has been identified [14, 15].

**Fig. 1:**
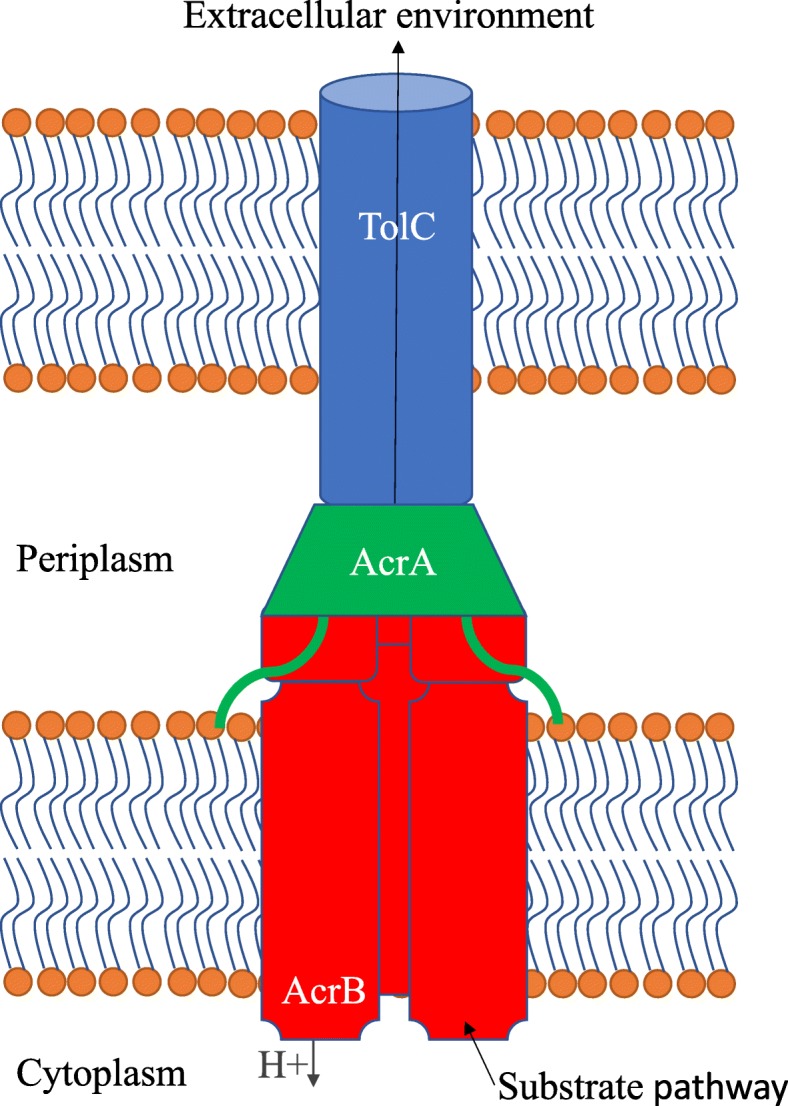
Schematic of the AcrAB-TolC multidrug efflux system as established in *E. coli*. The blue cylinder represents the TolC exit duct. The green trapezoid with two tails represents two AcrA adaptor proteins. The three red rectangles sans corners represent the AcrB homotrimer transporter protein

In *Escherichia coli*, the TolC outer membrane duct protein was originally identified to provide tolerance to colicin [16]. TolC was later shown to require other proteins to transport the intracellular substrates to the extracellular environment, providing tolerance to numerous antibiotics, dyes, and detergents [17]. The proteins necessary for the efflux of substrates using the *E. coli* TolC outer membrane duct are characterized into one of two groups: multidrug efflux pumps or membrane fusion proteins (MFPs). One example of a multidrug efflux pump is AcrB which works together with AcrA, an MFP, to export chemicals from within the *E. coli* cell out through the TolC duct [18, 19]. AcrB is responsible for transporting intracellular chemicals through the inner membrane, to the periplasmic opening of the TolC duct, while two AcrA proteins stabilize the opening of the TolC duct at the periplasmic end and bind to the homotrimer of AcrB [20–22]. The TolC duct allows for the substrates from the multidrug efflux pump to reach the extracellular environment passively [23] (Fig. 1). There are numerous other closely related multidrug efflux pumps and MFPs that are synthesized by *E. coli*, including EmrAB that has been shown to have overlapping function alongside AcrAB with respect to the efflux of FFAs; both AcrAB and EmrAB must be removed from *E. coli* to significantly decrease the efflux of FFAs [24]. All efflux pump systems possess the ability to actively extrude substrates from the cytoplasm through the lipid bilayer using the antiporter mechanism in which a proton is exchanged for the substrate in the multidrug efflux pump. Without any one of these three proteins, the tolerance to toxic chemicals that are substrates of the multidrug efflux system significantly decreases and resistance concomitantly increases in *E. coli* cells.

The TolC homolog in *Synechocystis* sp. PCC 6803, Slr1270, possesses a similar function to that of TolC in *E. coli* [14, 15]. Without Slr1270, the cyanobacteria possess a decreased tolerance to various antibiotics. In addition, *Synechocystis* sp. PCC 6803 that lacks a functional Slr1270 has a reduced ability to form biofilms and secrete outer membrane vesicles; the mutant strain also has a missing S-layer [15]. Slr1270 is thus predicted to be one component of the multidrug efflux mechanism for efflux of chemicals from *Synechocystis* sp. PCC 6803. There exist numerous putative proteins in *Synechocystis* sp. PCC 6803 that have been described as MFPs and multidrug efflux pumps [25–28]. However, there is a limited understanding of the multidrug efflux pump(s) and MFP(s) of *Synechocystis* sp. PCC 6803 that function together with Slr1270 to transport chemicals out of the cell. Evidence suggests that one multidrug efflux pump is Slr2131, while Sll0180 is one of the described MFPs [25, 28]. AcrA is created from 397 amino acids while Sll0180 is composed of 501 amino acids. When comparing the homology of the two proteins, the first 131 amino acids of Sll0180 share an 18% identity to AcrA and, similarly, nearly the last 262 amino acids of Sll0180 share a 19% identity to AcrA (Sll0180 amino acids 500 and 501 were not included in this identity analysis) [29]. AcrB share far more protein sequence homology to Slr2131 relative to the comparison between AcrA and Sll0180. Slr2131 has a protein sequence length of 1061 amino acids, while AcrB has 1049 amino acids. Amino acid 2 through 1017 of Slr2131 watched matched with the a 1016 amino acid length central region of AcrB for a 41% identity with no gaps in the alignment [29], indicating that the vast majority of the two proteins sequences share protein binding sites and transmembrane domains that a paramount to the functioning of each pump. Slr2131 contributes to the secretion of chloramphenicol (Cm) and ethidium bromide (EtBr) as evidenced by a decrease in tolerance to Cm and EtBr in *Synechocystis* sp. PCC 6803 with a portion of the *slr2131* gene deleted [30]. A *sll0180* mutant *Synechocystis* sp. PCC 6803 strain was previously shown to have a greater susceptibility to sodium dodecyl sulfate (SDS) and lacks the S-layer [30]. The research featured in this article provides further insight into the hypothesis that Slr2131 and Sll0180 are members of the multidrug efflux pump family and MFP family, respectively, and may function together with Slr1270 to transport toxic chemicals out of the cell. However, the roles of Slr2131 and Sll0180 may not be limited to the efflux of antibiotics and dyes; they may also have a role in efflux of FFAs. The homologous AcrA and AcrB proteins in conjunction with TolC in *E. coli* are responsible for efflux of FFAs in *E. coli* [24]. If the native mechanisms of efflux in *Synechocystis* sp. PCC 6803 are better understood this can lead to genetically modified cyanobacterial cells with a more efficient process for FFA production and the ensuing purification from the extracellular media.

The research featured in this article looks to further the understanding of both Slr2131 and Sll0180 in respect to their ability to promote the tolerance of *Synechocystis* sp. PCC 6803 to various antibiotics. SD100 is a single colony isolate of wild-type *Synechocystis* sp. PCC 6803 [31] and is the parental strain for all of the strains featured in this research. To begin the research, each of the SD100 genes, *slr2131* and *sll0180*, that encode the Slr2131 and Sll0180 proteins, respectively, were removed with replacement deletions from both SD100 and SD277, a strain derived from SD100 which contains numerous genetic modifications for the promotion of FFA production [8]. Previous researchers have hypothesized that SD277 uses the *flip-flop* method of diffusion of FFAs across the membrane [8], in which FFAs quickly diffuse across the lipid bilayer [32]. But we contend that there exists an active mechanism for FFA transportation to the extracellular environment, so the intracellular and extracellular FFA concentrations of SD100, SD277, and the deletion mutant strains were measured to identify that Slr2131 and Sll1080 are responsible for the secretion of FFAs. Then the mutations found in the deletion mutant strains derived from SD100 and SD277 were complemented with the homologous wild-type genes from *E. coli*, *acrB* or *acrA*, to counteract the deleterious effects of the native gene removals. The addition of the genes encoding the *E. coli* AcrB and AcrA proteins affects the secretion of FFAs and the tolerance to antibiotics in the SD100 and SD277 mutant strains. By further the understanding of active efflux in *Synechocystis* sp. PCC 6803, the cost and time associated with using the cyanobacteria for the manufacturing of chemicals can be reduced.

## Results

Sixteen strains based on either SD100 or SD277, the high FFA synthesizing strain, were created to investigate the roles of putative transporters in SD100 and SD277 and the ability to express homologous *E.* coli genes encoding the AcrA and AcrB proteins in both SD100 and SD277. A summary of all of the strains and the plasmids used to obtain the following results are provided (Table 1) along with the primers designed for the purposes of this research (Table 2).

**Table 1:** Strains and plasmids used in this study

	Description	Parent	Curtiss Collection Name	Reference
Strain Name				
SD100	Single colony isolate of wild-type Synechocystis sp. PCC 6803	N/A	SD100	30
SD277	High free fatty acid producing strain	SD100	SD277	8
SD100 △*slr2131*	SD100 with *slr2131* deleted	SD100	SD659	this research
SD277 △*slr2131*	SD277 with *slr2131* deleted	SD100	SD660	this research
SD100 △*sll0180*	SD100 with *sll0180* deleted	SD277	SD661	this research
SD277 △*sll0180*	SD277 with *sll0180* deleted	SD277	SD662	this research
SD100 △*slr2131* p*acrB*	SD100 with *slr2131* deleted with expression vector expressing *E. coli acrB*	SD100 △*slr2131*	SD665	this research
SD277 △*slr2131* p*acrB*	SD277 with *slr2131* deleted with expression vector expressing *E. coli acrB*	SD277 △*slr2131*	SD666	this research
SD100 △*sll0180* pS*acrA*	SD100 with *sll0180* deleted with expression vector expressing *E. coli acrA*	SD100 △*sll0180*	SD667	this research
SD277 △*sll0180* pS*acrA*	SD277 with *sll0180* deleted with expression vector expressing *E. coli acrA*	SD277 △*sll0180*	SD668	this research
SD100 p*acrB*	SD100 p⍦691 (Ec *acrB)*	SD100	SD643	this research
SD277 p*acrB*	SD277 p⍦691 (Ec *acrB)*	SD277	SD644	this research
SD100 p*acrA*	SD100 p⍦692 (Ec *acrA)*	SD100	SD645	this research
SD277 p*acrA*	SD277 p⍦692 (Ec *acrA)*	SD277	SD646	this research
SD100 pSpec	SD100 with empty expression vector conferring Spc resistance	SD100	SD703	this research
SD277 pSpec	SD277 with empty expression vector conferring Spc resistance	SD277	SD704	this research
SD100 pKan	SD100 with empty expression vector conferring Kan resistance	SD100	SD705	this research
SD277 pKan	SD277 with empty expression vector conferring Kan resistance	SD277	SD706	this research
Plasmid Name				
pGEM-3Z	commercially available cloning vector	N/A	N/A	N/A
p⍦694	vector pGEM-3Z harboring *slr2131* flanking regions with *sacB-*Kan^R^ cassette inserted between	pGEM-3Z	p⍦694	this research
p⍦695	vector pGEM-3Z harboring *sll0180* flanking regions with *sacB-*Kan^R^ cassette inserted between	pGEM-3Z	p⍦695	this research
pSpec	Expression vector conferring resistance to streptomycin and spectinomycin	RSF1010	p⍦568	this research
pKan	Expression vector conferring resistance to kanamycin	RSF1010	p⍦687	this research
p*acrB*	Expression vector expressing *E. coli acrB*	pSpec	p⍦691	this research
pS*acrA*	Expression vector expressing *E. coli acrA*	pSpec	p⍦701	this research
p*acrA*	Expression vector expressing *E. coli acrA*	pKan	p⍦692	this research

**Table 2:** List of the primers used to create the plasmid and strains and primers used for RT-PCR analysis

	Sequence (5′-3)	Reference
Primer Name		
A 3Z slr 2131 end F	GAG AGA GCT CGA CCC CGT TGT GTT AA	this research
A 3Z slr 2131 center R	GAT TTA TTT TCT TGG ATC CCA TTA CGG CCG ACA TTG TTA CAT ATC T	this research
A 3Z sll 0180 end F	CCT GGA GCT CTT GAC AAT GAC GAC AAT C	this research
A 3Z sll 0180 center R	TTT TTA GTT CTG GAT CCC ATT ACG GCC GTA GTT AAT TGA CTC A	this research
B 3Z slr 2131 center F	TGT AAC AAT GTC GGC CGT AAT AGG ATC CAA GAA AAT AAA TCT GGT CTT ATT	this research
B 3Z slr 2131 end R	GCG CGT CTA GAT ATC CCA GTT CCA TTC TTT G	this research
B 3Z sll 0180 center F	TGA GTC AAT TAA CTA CGG CCG TTG GAT CCA GAG AAC TAA AAA GTC TA	this research
B 3Z sll 0180 end R	CGG GTC TAG ACT GCT GGG CAT TAT C	this research
Kan BamHI	CAT TAC ACC AAG GAA TTA GGA TCC GTC GAC C	this research
EagI SacB	ATA TCG GCC GGA ACA TCG ACA AAT ACA TAA GGA AT	this research
5’ AcrB F	CGA TGG ATC CAG TCT TAA CTT AAA CAG GAG C	this research
3’ AcrB R	AAT TGC ATG CAT AAA AAA GGC CGC TTA CGC	this research
3’ AcrA R	ATA TGC ATG CAC GGC TCC TGT TTA AGT TAA	this research
5’ AcrA F	GCACGGATCC TTACATATGAACAAAAACAGAGG	this research
2131 w/in A	CCA CTT CTT TGG TAT TGA TGG CAG	this research
2131 w/in B	GGA GCT TGG ATA ATG GTG ATG AAA TAA	this research
Primers for RT-PCR		
*petB*-F	CCTTCGCCTCTGTCCAATAC	55
*petB-*R	TAGCATTACACCCACAACCC	55
acrA-F	CTCTCAGGCAGCTTAGCCCTAA	54
acrA-R	TGCAGAGGTTCAGTTTTGACTGTT	54
acrB-F	GGTCGATTCCGTTCTCCGTTA	54
acrB-R	CTACCTGGAAGTAAACGTCATTGGT	54

### The removal of *slr2131* or *sll0180* generally decreases tolerance to antibiotics

After deleting either *slr2131* or *sll0180* from both SD100 and SD277, the maximal half inhibitory concentration (IC_50_) was determined when each strain was exposed to the antibiotics ampicillin (Amp), chloramphenicol (Cm), or Erythromycin (Ery) at concentrations between 10 ng/mL and 100 mg/mL (Table 3). SD100 Δ*slr2131* possessed significant decreases in tolerance to Cm and Ery at 74 and 85%, respectively, when compared to the wild-type SD100. However, when SD100 Δ*slr2131* was treated with Amp, there was no significant difference when compared to wild-type SD100*.* SD277 Δ*slr2131* had significant decreases in tolerance to Amp, Cm, and Ery of 36, 61, and 60%, respectively when compared to SD277. SD100 Δ*sll0180* possessed significant decreases in tolerance when treated with Amp, Cm, and Ery of 37, 37, and 52%, respectively, when compared to SD100. SD277 Δ*sll0180* possessed significant decreases in tolerance to Amp, Cm, and Ery of 71, 77, and 92%, respectively, when compared to SD277.

**Table 3:** IC_50_ of each parent, mutant derivative, and complementation derivative strain and the percent differences of tolerance relative to the parent strain

Treatment		SD100	SD100 Δ*slr2131*	SD100 Δ*slr2131* p*acrB*	SD100 Δ*sll0180*	SD100 Δ*sll0180* pS*acrA*
Amp	IC_50_	955.2 ± 256 μg/mL	1280 ± 230 μg/mL (ns)	891 ± 117 μg/mL (ns)	598 ± 296 μg/mL	577.0 ± 23.2 μg/mL
	% diff from SD100	0%	34%	−6.7%	37%	39%
Cm	IC_50_	2.290 ± 0.401 μg/mL	0.586 ± 0.142 μg/mL	2.124 ± 1.004 μg/mL (ns)	1.442 ± 0.206 μg/mL	4.73 ± 1.47 μg/mL
	% diff from SD100	0%	−74%	−7.2%	−37%	106%
Ery	IC_50_	0.358 ± 0.081 μg/mL	0.054 ± 0.002 μg/mL	0.103 ± 0.019 μg/mL	0.172 ± 0.1 μg/mL	0.144 ± .028 μg/mL
	% diff from SD100	0%	−85%	−71%	−52%	−60%
Treatment		SD277	SD277 Δ*slr2131*	SD277 Δ*slr2131* p*acrB*	SD277 Δ*sll0180*	SD277 Δ*sll0180* pS*acrA*
Amp	IC_50_	1570 ± 170 μg/mL	1001 ± 120 μg/mL	980.3 ± 37 μg/mL	450 ± 119 μg/mL	286.6 ± 113.8 μg/mL
	% diff from SD277	0%	−36%	−38%	−71%	−82%
Cm	IC_50_	4.749 ± 1.043 μg/mL	1.873 ± 0.354 μg/mL	3.305 ± 1.100 μg/mL (ns)	1.085 ± 0.589 μg/mL	3.486 ± 1.027 μg/mL (ns)
	% diff from SD277	0%	−61%	−30%	−77%	−27%
Ery	IC_50_	0.427 ± 0.249 μg/mL	0.171 ± 0.044 μg/mL	0.095 ± 0.007 μg/mL	0.034 ± 0.007 μg/mL	0.075 ± 0.014 μg/mL
	% diff from SD277	0%	−60%	−78%	−92%	−82%

The basis for the comparison in this table were the parent strains, either SD100 or SD277, featured in the third column. The data values for each derivative strain are significantly different from the parent strain unless otherwise noted with “(ns)”

### The addition of the *E. coli acrB* gene to the cyanobacterial mutants lacking *slr2131* and the addition of the *E. coli acrA* gene to mutants lacking *sll0180* restored tolerance to chloramphenicol

SD100 Δ*slr2131* possessed a 74% decrease in tolerance to Cm when compared to SD100, but when the *E. coli acrB* gene was introduced on an expression vector (p*acrB*) into the deletion mutant, the resulting strain, SD100 Δ*slr2131* p*acrB*, no difference of tolerance to Cm when compared to SD100 indicating that the original tolerance was restored (Table 3). SD277 Δ*slr2131* p*acrB* and SD277 Δ*sll0180* pS*acrA* each had a significant increase in tolerance to Cm compared to each’s respective mutant parent strain, while each have an equivalent IC_50_ to Cm when compared SD277. SD100 Δ*sll0180* pS*acrA* had an increased tolerance to Cm with a significant 106% increase when compared to SD100; SD100 Δ*sll0180* pS*acrA* also possessed a significant 228% increase in tolerance to Cm when compared to SD100 Δ*sll0180*. The addition of *E. coli acrB* to SD100 Δ*slr2131* or SD277 Δ*slr2131* did not restore the tolerance to Amp and Ery to equivalent concentrations observed in each mutant strain’s respective parent strain, SD100 and SD277. The addition of *E. coli acrA* to SD100 Δ*sll0180* or SD277 Δ*sll0180* did not restore the tolerance to Amp or Ery to equivalent concentrations observed in each mutant strain’s respective parent strain, SD100 and SD277. Outside of the restoration of tolerance to Cm, there does not exist any evidence that the addition of *E. coli acrA* or *acrB* compensate for the loss of tolerance due to the deletion of native *sll0180* or *slr2131* from SD100 and SD277.

### The removal of either *slr2131* or *sll0180* from SD277 and SD100 modifies the extracellular and intracellular FFA concentrations

**Fig. 2:**
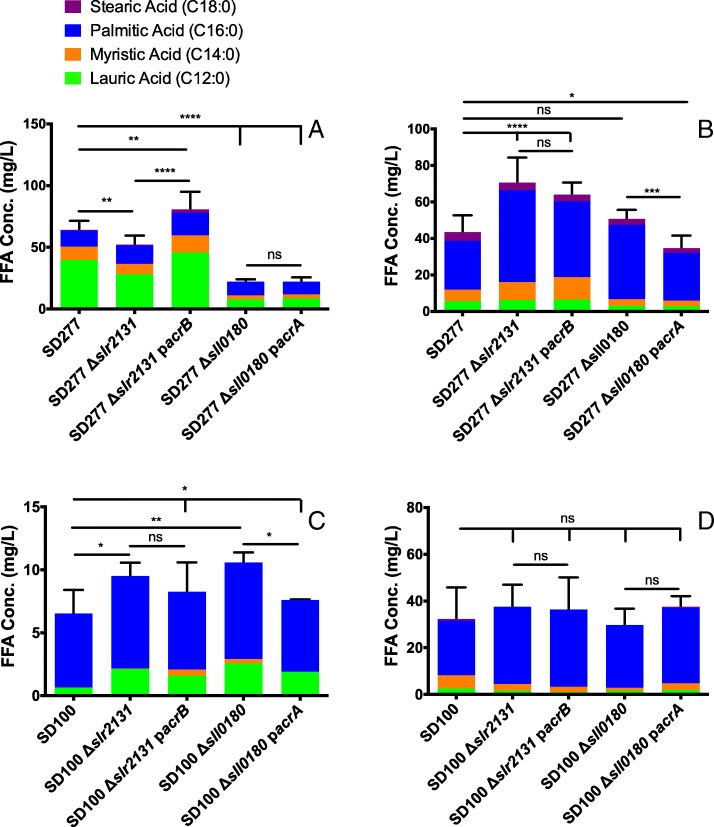
FFA concentration of SD277, mutant, and complementation derivatives (**a**) extracellularly and (**b**) intracellularly. FFA concentration of SD100 and its mutant and complementation derivatives (**c**) extracellularly and (**d**) intracellularly in which * is *p* < 0.05, ** is *p* < 0.005, *** is *p* < 0.0005, and **** is *p* < 0.0001. Each color represents an average concentration of a saturated fatty acid: green represents lauric acid, orange represents myristic acid, blue represents palmitic acid, and purple represents stearic acid

The total concentrations of extracellular FFA of SD277 Δ*slr2131* and SD277 Δ*sll0180* were significantly less than SD277 with differences of 19 and 65% respectively (Fig. 2a). These were proportional changes in concentrations from each of the 4 measured FFAs. The intracellular FFA concentrations of SD277 Δ*slr2131* and SD277 Δ*sll0180* both had increases in total concentration when compared to SD277, with differences of 62 and 17% respectively (Fig. 2b); only the 62% increase was significant. Both SD100 Δ*slr2131* and SD100 Δ*sl10180* possessed significant increases in extracellular FFA concentrations when compared to SD100, but while statistically significant, the actual values of the decreases were between 3 and 4 mg/L of total FFA concentrations (Fig. 2c). These values represent one log level less than the amount difference values of the SD277 mutant strains compared to SD277. There were no significant differences when comparing the intracellular FFA concentrations of SD100 to SD100 Δ*slr2131* and SD100 Δ*sll0180* (Fig. 2d).

### The addition of the *E. coli acrB* to the mutant SD100 and SD277 strains lacking *slr2131* and the addition of the *E. coli acrA* gene to mutant strains lacking *sll0180* modified the extracellular and intracellular FFA concentrations

SD277 Δ*slr2131* p*acrB*, possessed a significant increase in the concentration of extracellular FFAs when compared to SD277 and SD277 Δ*slr2131* with an increase of 26 and 55% respectively, while the intracellular FFA concentration of SD277 Δ*slr2131* p*acrB* was no different to that of SD277 Δ*slr2131* (Fig. 2a-b). When *E. coli acrA* was added to SD277 Δ*sll0180*, hereafter referred to as SD277 Δ*sll0180* pS*acrA*, the extracellular FFA concentration was the same as SD277 Δ*sll0180*, maintaining a significantly lower concentration of FFAs when compared to SD277, while the intracellular concentration of SD277 Δ*sll0180* pS*acrA* was significantly lower than SD277 Δ*sll0180* (Fig. 2a-b). The addition of the *E. coli acrB* gene to SD100 Δ*slr2131* (SD100 Δ*slr2131* p*acrB*) did not change in the extracellular or intracellular FFA concentrations of the strain when compared to SD100 Δ*slr2131* (Fig. 2c-d). The addition of *E. coli acrA* to SD100 Δ*sll0180*, now denoted as SD100 Δ*sll0180* pS*acrA*, significantly lowered the concentration of the extracellular FFAs when compared to SD100 Δ*sll0180*, but still maintained a significantly higher concentration of FFA when compared to SD100 (Fig. 2c). Again, the changes in concentrations of the SD100 derivative strains were under the 4 mg/L, a log level lower than the changes observed in the corresponding SD277 and SD277 derivative strains. There were no significant differences among the concentrations of intracellular FFAs when comparing SD100 Δ*sll0180* pS*acrA* and SD100 Δ*slr2131* p*acrB* to SD100 or the SD100 derivative mutant strains (Fig. 2d).

### Identifying the roles of *E. coli* genes and expression vectors have on FFA efflux and IC_50_

**Fig. 3:**
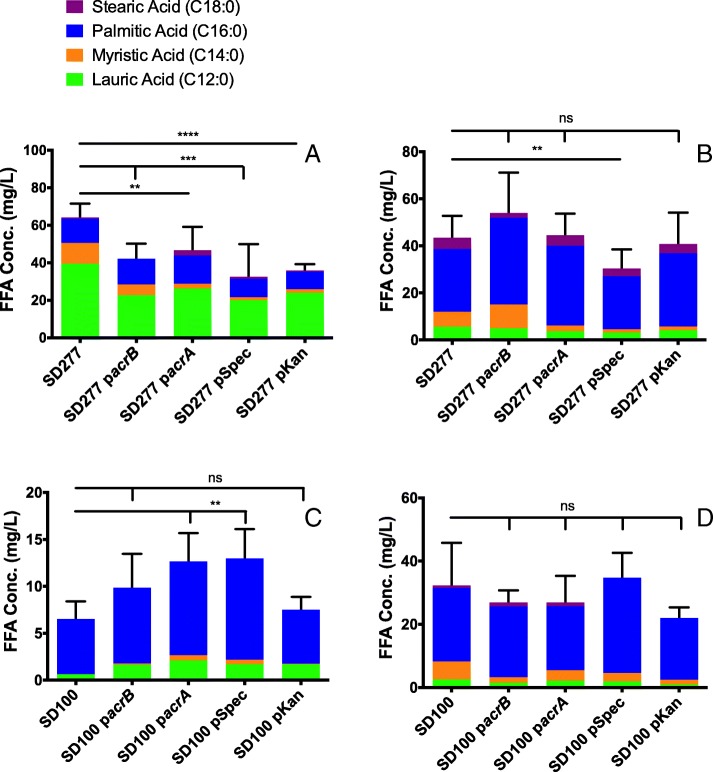
FFA concentration of SD277 and its derivatives (**a**) extracellularly and (**b**) intracellularly. FFA concentration of SD100 and its derivatives (**c**) extracellularly and (**d**) intracellularly in which * is *p* < 0.05, ** is *p* < 0.005, *** is *p* < 0.0005, and **** is *p* < 0.0001. Each color represents an average concentration of a saturated fatty acid: green represents lauric acid, orange represents myristic acid, blue represents palmitic acid, and purple represents stearic acid

To identify what role the *E. coli acrA* and *acrB* genes have in SD277 and SD100, p*acrA* and p*acrB* were added to SD100 and SD277 individually. In addition, the expression vectors lacking these two *E. coli* genes, pKan and pSpec, were each added individually to identify if the plasmid has a role in chemical efflux. When either p*acrA* or p*acrB* was added to SD277, the resulting strains, SD277 p*acrA* and SD277 p*acrB*, had a significant decrease in the FFA concentrations extracellularly when compared to SD277 (Fig. 3a). This was the same case with respect to the strains resulting from the addition of pKan and pSpec to SD277, SD277 pKan and SD277 pSpec (Fig. 3a). With respect to intracellular FFA concentrations, when p*acrA*, p*acrB*, or pKan were added individually to SD277 none of the resulting strains had a significant difference when compared to SD277 (Fig. 3b). However, when pSpec was added to SD277, the SD277 pSpec strain had a significant decrease of FFA concentration intracellularly (Fig. 3b). SD100 p*acrA* and SD100 pSpec each had a significantly higher concentration of FFA extracellularly while SD100 p*acrB* and SD100 pKan did not vary significantly when compared to SD100 (Fig. 3c). When the concentrations of intracellular FFAs were measured, none of the SD100 based strains with the added plasmids were significantly different when compared to SD100 (Fig. 3d).

With respect to the IC_50_ of the resulting strain when any of the four plasmids (p*acrA*, p*acrB*, pKan, pSpec) were added to SD100 or SD277, the plasmids largely either had an effect to decrease or provided no change to the IC_50_ relative to the parent strain (Table 4). The one exception was SD277 pKan when treated with Ery; this strain had a slight, yet significant increase in IC_50_ when compared to SD277.

**Table 4:** IC_50_ of each parent and plasmid addition strain and percent differences of tolerance relative to the parent strain

Treatment		SD100	SD100 p*acrA*	SD100 pKan	SD100 p*acrB*	SD100 pSpec
Amp	IC_50_	955.2 ± 256	1315 ± 77 (ns)	1151 ± 82 (ns)	1109 ± 123 (ns)	658.6 ± 88.3
	% diff from SD100	0%	27%	20%	16%	−31%
Cm	IC_50_	2.290 ± 0.401	1.444 ± 0.114	1.390 ± 0.186	1.976 ± 0.734 (ns)	1.653 ± 0.205 (ns)
	% diff from SD100	0%	−59%	−39%	−14%	−28%
Ery	IC_50_	0.358 ± 0.081 μg/mL	0.080 ± 0.003	0.072 ± 0.007	0.138 ± 0.080 (ns)	0.079 ± 0.005
	% diff from SD100	0%	−77%	− 79%	−61%	−78%
Treatment		SD277	SD277 p*acrA*	SD277 pKan	SD277 p*acrB*	SD277 pSpec
Amp	IC_50_	1570 ± 170 μg/mL	1117 ± 21 μg/mL	76.67 ± 28.5 μg/mL	118.2 ± 8.7 μg/mL	412.6 ± 34.8 μg/mL
	% diff from SD277	0%	−29%	−95%	−92%	−74%
Cm	IC_50_	4.749 ± 1.043 μg/mL	1.833 ± 0.390 μg/mL	1.793 ± 0.430 μg/mL	1.984 ± 0.527 μg/mL	2.96 ± 0.4 μg/mL
	% diff from SD277	0%	−61%	− 62%	−58%	−38%
Ery	IC_50_	0.427 ± 0.249 μg/mL	0.091 ± 0.034 μg/mL	0.149 ± 0.016 μg/mL	0.080 ± 0.008 μg/mL	0.055 ± 0.004 μg/mL
	% diff from SD277	0%	−79%	−65%	−81%	−87%

The basis for the comparison in this table were the parent strains, either SD100 or SD277, featured in the third column. Each derivative strain is significantly different from the parent strain unless otherwise noted with “(ns)”

### mRNA and growth rates

To ensure that the *E. coli* genes were expressing the DNA that was present in the constitutively expressing promoters located on the plasmids, the mRNA was purified, and cDNA was created using reverse-transcriptase PCR (RT-PCR). In each case in which the *E. coli* gene was added to a cyanobacterial strain, a corresponding cDNA sequence was identified using gel electrophoresis, with none of the *E. coli* genes identified in parent, mutant, or vector control strains (Additional file 1: Figure S1A**)**. Each cyanobacterial strain was also tested for the presence of *petB* mRNA, a constitutively expressed cyanobacterial gene. mRNA of *petB* was identified in each cyanobacterial sample (Additional file 1: Figure S1B). A conventional PCR was performed using the identical primers used for the RT-PCR to verify that there was no genomic cyanobacterial DNA contamination detected in the mRNA samples after the DNase treatment (Data not shown) (Table 2).

To understand the growth pattern of the cyanobacteria, each strain was grown for 168 h in triplicate beginning at an OD_730_ of 0.05. There were no differences in any of the growth phases among any strain presented in this research when compared to its respective parent strain, either SD100 or SD277 (Additional file 1: Figure S2A-D).

## Discussion

Considering the numerous advancements using genetically modified bacterial strains based on *Synechocystis* sp. PCC 6803 for production of biofuels and other industrially relevant chemicals, there is a limited understanding regarding the transportation of these chemicals out of the cyanobacterial cells. This current research looked to corroborate the hypothesis that *Synechocystis* sp. PCC 6803 genes *slr2131* and *sll0180* encode homologous proteins to that of *E. coli* AcrB and AcrA, respectively. To do this, this research presents three hypotheses. The first is that the removal of *slr2131* or *sll0180* from both SD100 and SD277 will inhibit the cell’s ability to tolerate antibiotics and decrease the concentrations of extracellular FFAs while increasing intracellular FFA concentrations. Second, adding the homologous *E. coli acrA* and *acrB* genes to the SD100 and SD277 *sll0180* and *slr2131* mutant strains will restore the lost tolerance to Amp, Cm, and Ery; additionally, extracellular FFA concentrations will be increased. And, the final hypothesis addressed was that adding the vectors containing the *E. coli acrA* or *acrB* genes to SD100 and SD277, will increase the tolerance to Amp, Cm, and Ery, and increase the extracellular FFA concentrations.

This research showed that removing *sll0180* and *slr2131* from SD100 and SD277 significantly decreased tolerance to Amp, Cm, and Ery. Both SD100 and SD277 rely on the encoded proteins, Sll0180 and Slr2131, for efflux of these chemicals and without either Sll0180 or Slr2131, the tolerance decreases. Previous research performed by Gonçalves et al. (2018) supports the hypothesis that Sll0180 is necessary for native efflux in that the removal of *sll0180* from *Synechocystis* sp. PCC 6803 caused a significant inhibition of growth due to the presence of Cm [30]. However, Gonçalves et al. (2018) did not observe a significant inhibition of growth associated with the removal of *slr2131* from *Synechocystis* sp. PCC 6803 due to the presence of Cm, as shown in the currently presented research. With respect to the *sll0180* and *slr2131* mutants in *Synechocystis* sp. PCC 6803, Gonçalves et al. (2018) did also associate a significant growth inhibition when treated with 0.0025% SDS [30], providing further support that both of the encoded proteins confer part of an efflux mechanism. In addition, *Synechocystis* sp. PCC 6803 does not appear to have another efflux mechanism capable of substituting for the efflux roles that Sll0180 or Slr2131 proteins confer to the cyanobacteria. The *E. coli* AcrB multidrug efflux pump has other homologous pumps encoded by the *E. coli* genome, AcrD and AcrF, that have some identical substrates to AcrB [33]. However, *Synechocystis* sp. PCC 6803 does not appear to have this redundancy as indicated by the significant decrease in tolerance to Amp, Cm, and Ery, when *slr2131* was removed from the *Synechocystis* sp. PCC 6803 chromosomes. If another protein existed that possessed a similar to either Sll0180 or Slr2131 then there would likely not be a significant decrease in the tolerance to the various chemicals of all of the mutant strains; additionally, the extracellular FFA concentration of the two SD277 mutant strains would also not be decreased significantly. The idea of redundant proteins used in transportation of chemicals is explored subsequently with respect to the *E. coli* EmrAB and AcrAB multidrug efflux pumps.

When either of the native *slr2131* or *sll0180* genes were removed from the high FFA producing SD277 strain, the results validate the hypothesis that both of the conferred proteins, Slr2131 and Sll0180, contribute to the secretion of FFAs from within the SD277 cell to the extracellular environment. When either gene was deleted, the extracellular FFA concentrations decreased significantly, while the FFA concentrations within the cell increased. This parallels the results observed when *acrAB* was removed concurrently with *emrAB*, a distinct multidrug efflux system, from *E. coli*. Lennen et al. (2013) caused a significant drop in the extracellular FFA concentration in *E. coli* that was genetically engineered to produce FFAs at concentrations 450% higher than wild-type *E. coli* [24]. Removing the *acrAB* operon alone from the high FFA producing *E. coli* strain did not create a strain that had a significantly lower extracellular FFA concentration than the high FFA producing *E. coli* strain with native *acrAB* present. FFAs are substrates of both *E. coli emrAB* and *acrAB* [34]. Conversely in the cyanobacterial strain SD227, because the removal of just a single gene, e.g. *slr2131*, from SD277 caused a significant decrease extracellular FFA concentration, there is no apparent homologous protein to Slr2131 in SD277 that can transport FFAs out of the cell in a similar manner to the complementary FFA efflux function conferred by the *E. coli* EmrB multidrug efflux pump when the high FFA producing *E. coli* strain does not have AcrB. *Synechocystis* sp. PCC 6803 has approximately one-third of the total transporters that *E. coli* does, approximately 100 compared to 300 total transporters may contribute to the lack of pumps with overlapping function in the cyanobacteria [35]. 5% of the *E. coli* genome encodes approximately 79 ATP-Binding Cassette (ABC) transporters [36]. This is compared to the 54 putative and identified ABC transporters of *Synechocystis* species [37]. This large discrepancy in the number of transporters is likely to do the dramatically different environments that *E. coli* and *Synechocystis* sp. PCC 6803 evolved throughout the millennia. But genetically modifying SD100 and SD277 can alter the results of millennia of evolution to assist in the production of precursors to biofuels.

When *E. coli acrA* was added to SD100 and SD277 strains without *sll0180* or *E. coli acrB* was added to SD100 and SD277 strains without *slr2131*, the only consistent restoration of tolerance when compared to the parent strains was to Cm. Tolerance to Ery and Amp was inconsistent across the four complementation strains with examples that had decreased, increased, and statistically identical IC_50_ relative to the corresponding mutant strain. But none of the complementation strains had IC_50_ statistically the same or greater than the parent strain when treated with increasing concentration of Ery and Amp. Cm, Amp, and Ery are all established substrates of AcrB [18, 38, 39], with the *E. coli* tolerance to each chemical usually decreasing especially if other multidrug efflux systems are removed concurrently. So, it’s not clear why Cm is readily exported in *Synechocystis*-derivative strains while the other 2 antibiotics are not when the *E. coli* genes are expressed. However, Cm is a polar compound while Amp and Ery are not. Cm contains two N-O polar bonds in a bent conformation. There are 11 polar residues in *E. coli* AcrB that contribute to binding of substrates [40], which may allow for Cm to be bound preferentially to the nonpolar Ery and Amp.

The export of chemicals was not limited to antibiotics; when *acrB* was expressed in SD277 Δ*slr2131* mutants, FFA concentration was significantly increased extracellularly compared to both SD277 and SD277 Δ*slr2131*. SD277 Δ*slr2131* p*acrB* had a greater intracellular FFA concentration than SD277 but statistically equivalent to SD277 Δ*slr2131*. We expected the intracellular FFA concentration of SD277 Δ*slr2131* p*acrB* to have a statistically significant decrease relative to SD277 Δ*slr2131* as the addition of p*acrB* led to an increase in extracellular FFAs, but we instead observed a decreasing trend in intracellular FFA concentration. This is one example of unexpected results between extracellular and intracellular FFA concentrations. This may be attributed to CO_2_ limitations in the production of FFAs. If we had increased the CO_2_ concentration in the aeration of the samples, these trends may have had significant differences as the total FFA production would have increased [41]. However, in this research we determined the process for moving FFAs out of the cell is not a result of diffusion; the extracellular and intracellular concentrations may not be directly proportional. Furthermore, AcrB is able to transport FFAs out of the cell in *Synechocystis*-based strains. AcrB likely does not act alone and may use Slr1270 as a partner to transport the FFA out of the cell. Slr1270 has previously been shown to have similar physical characteristics to TolC when *slr1270* is placed in *E. coli* [14]. Furthermore, when *E. coli acrB* is added to SD277, there is no increased extracellular FFA concentration in the resulting SD277 p*acrB* strain when compared to SD277. The native SD277 Slr2131 protein may be competitive with *E. coli* AcrB protein synthesized in SD277 for outer membrane ducts, such as Slr1270. Once Slr2131 is removed, constitutively synthesized *E. coli* AcrB continuously transports FFAs out of the SD277 Δ*slr2131* p*acrB* cells; although, it is not clear if AcrB needs an MFP to facilitate the transportation of FFA out of the SD277 Δ*slr2131* p*acrB* strain. The FFAs located intracellularly of SD277 Δ*slr2131* p*acrB* remain significantly higher than SD277 and SD277 Δ*slr2131*, so the addition of p*acrB* to SD277 Δ*slr2131* does not alleviate the increased intracellular FFA concentration observed in SD277 Δ*slr2131*. But the SD277 Δ*slr2131* p*acrB* cells tolerate the elevated concentrations as their growth rates do not vary significantly from SD277 (Additional file 1: Figure S2A). There are a few genetic manipulations to SD100 to modify the cell membrane structure including the removal of *sll1951* and *slr1710* that led to the creation SD277. Sll1951, the protein encoded by *sll1951*, is necessary for the native structure of the S-layer of the cyanobacteria to be present while *slr1710* encodes a penicillin binding protein necessary for the peptidoglycan layer assembly [42]; these two modification to the cell membrane were an effort to allow FFAs to flow out of the cell with less encumbrance. The addition of *E. coli* AcrB to SD277 Δ*slr2131* increases the extracellular FFA concentration relative to SD277, indicating that the strategy of active transportation of FFA out of the cyanobacterial cell is a more efficient strategy than eliminating cell membrane proteins to move FFA out of the cell.

The addition of *acrA* to SD277 Δ*sll1080* did not provide any increase in FFA concentration extracellularly. *E. coli* AcrA may just not effectively recognize binding sites on the Slr1270 outer membrane duct or there may exist differences in the membrane structure of these cyanobacteria that may not be ideal or even necessitate AcrA. When aligning the 397 amino acids of AcrA and the 501 amino acids of Sll0180, there exists a gap from amino acids 132 to 237 of Sll0180, bearing virtually no homology to AcrA. When identifying possible protein binding regions in these 105 nonhomologous amino acids of Sll0180, there are two are predicted at sites 132 and 183. AcrA does not possess this central region and therefore these two predicted protein binding sites that are present in Sll0180. The lack of this central region may affect the ability of AcrA to bind to a protein within the membranes of *Synechocystis* sp. PCC 6803. In addition, previous research has supported the hypothesis that the periplasmic end of the Slr1270 outer membrane duct in *Synechocystis* sp. PCC 6803 remains open without the MFP, which is not the case for *E. coli* TolC lacking an MFP. Without *E. coli* AcrA or a different MFP, the periplasmic end of *E. coli* TolC collapses, while Slr1270 maintains its open at both ends of the duct without an MFP [14]. Based on the results of the *sll0180* mutant strains, the Sll0180 MFP is necessary for a native efflux mechanism, but the resulting loss of function cannot be compensated through the addition of *E.* coli AcrA in SD100 or SD277 *sll0180* mutant strains. The compounding differences between Sll0180 and AcrA including the amino acid sequence lengths (501 compared to 397), nonhomologous protein binding sites, and the different roles each may have in relation to the outer membrane duct may all lead to the inability for *E. coli* AcrA to restore the loss of function demonstrated in *sll0180* mutant SD100 and SD277 strains with respect to FFA secretion and tolerance to Amp, Cm, and Ery.

Finally, the four vectors, pKan, pSpec, p*acrA*, and p*acrB* were transferred into the parent strains, SD100 or SD277, to understand the role that the addition of these plasmids had on tolerance to chemicals and FFA concentration, intracellularly and extracellularly. This resulted in SD100 derivative strains with universally decreased or unchanged tolerance to the 3 chemicals tested. However, the SD277 derivative strains featured strains that that were observed to have increased, decreased, and statistically unchanged tolerances to the 3 chemicals tested when compared to SD277, making any broad conclusions about the effect from the plasmids on the tolerance of SD277 to the various chemicals difficult to corroborate with the results presented in this research. Additionally, SD277 pSpec was observed to have a significantly lower intracellular and extracellular FFA concentration than SD277, while SD277 pKan had a lower extracellular FFA concentration but statistically identical intracellular FFA concentration to SD277. The observed decrease in the intracellular FFA concentration of SD277 pSpec, which may be the cause of the observed significant decrease in the extracellular FFA concentration provided evidence contrary to the hypothesis that the addition of the control expression vector would not change the extracellular or intracellular FFA concentrations of SD277. In numerous bacteria including *Bacillus subtilis* and *Vibrio cholerae*, the inducible SOS DNA repair system is modulated, in part, by LexA that functions as a repressor. Once DNA damage occurs, ssDNA is formed which binds to RecA. This binding cleaves the LexA dimer that functions as a repressor to the SOS system, initiating DNA repair [43]. Additionally, LexA in *V. cholerae* has been shown to induce the SOS DNA repair response during bacterial conjugation [44]. Interestingly, *Synechocystis* sp. PCC 6803 possesses a *lexA* homolog, *sll1626*, and the encoded Sll1626 protein has a role in down-regulating *fab* (fatty acid biosynthesis) genes in *Synechocystis* sp. PCC 6803. The same down-regulation was observed in a modified strain of *Synechocystis* sp. PCC 6803 created to increase FFA synthesis [45]. The LexA homolog found in *Synechocystis* sp. PCC 6803, Sll1626, unlike that in *E. coli* and *V. cholerae*, is not linked to any genes involved in DNA metabolism in SD100 [46–48]. Sll1626 may not be involved in SOS DNA repair in *Synechocystis* sp. PCC 6803; however, Sll1626 still may be induced by bacterial conjugation and may be decreasing fatty acid biosynthesis in SD277 when p*acrA*, p*acrB*, pKan, or pSpec were added to a cyanobacterial strain. This downregulation of *fab* genes may be causing this significant decrease in extracellular and intracellular FFA concentrations observed in SD277 pSpec. A future study removing *sll1626* from the strains created for this research may help illustrate a picture of Sll1626 involvement in fatty acid biosynthesis and may help explain the decrease in FFA concentrations in SD277 strains containing exogenous plasmids.

While the addition of p*acrB* to SD277 decreased the extracellular FFA concentration of SD277, the result appears to be due to the addition of the plasmid to the SD277 strain. But this research has established the efficacy of the addition p*acrB* to the SD277 Δ*slr2131* strain, significantly increasing the extracellular FFA relative to both SD277 and SD277 Δ*slr2131*. This provides corroborating data to the hypothesis that the addition of the *E. coli acrB* gene to SD277 Δ*slr2131* increases the extracellular FFA concentration. This conclusion leads to the hypothesis that adding all three of the *E. coli* genes encoding the *E. coli* AcrAB-TolC multidrug efflux system to SD277 may further increase FFA concentration extracellularly. But removing the homologous *Synechocystis* sp. PCC 6803 genes, *sll0180*, *slr2131*, and *slr1270*, first from SD277 may assist in the function of the *E. coli* multidrug efflux pump system, considering that SD277 Δ*slr2131* p*acrB* had the highest concentration of FFA in this research. Additionally, without first understanding if bacterial conjugation modifies the expression of *sll1626*, the *lexA* homolog, which effects the resulting *fab* genes, *E. coli* gene additions should be placed in the genome. The negative phenotypes observed in this research due to the addition of the vectors may be avoided by placing the *E. coli* genes in the cyanobacterial chromosome.

## Conclusions

In conclusion, the data presented illustrates the role of the *Synechocystis* sp. PCC 6803 proteins encoded by *slr2131* and *sll0180* in chemical efflux. Without each one of these genes present, the cyanobacteria have decreased tolerance to antibiotics. Additionally, extracellular FFA concentrations are decreased, while intracellular FFA are increased in SD277 mutant strains. *E. coli* homologs of *slr2131* and *sll0180*, *acrA* and *acrB*, were expressed in SD100 and SD277 and created distinct phenotypes including the extracellular FFA concentration increase of the SD277 Δslr2131 p*acrB* strain, producing the highest concentration of extracellular FFA observed in this research. This research continues the understanding of chemical transportation through the cell membrane of *Synechocystis* sp. PCC 6803 and introduces the ability to use *E. coli* transport genes to alter the native efflux mechanism.

## Methods

### Bacterial strains, plasmids, growth conditions, and reagents

Bacterial strains and plasmids used in this study are listed in Table 1. For routine use, *Synechocystis* sp. PCC 6803 was inoculated on a BG-11 agar plate [49] and grown in illumination (50 μmol photons m^− 2^ s^− 1^) at 30 °C in an environmental chamber (SANYO, Osaka, Japan). Strains with the *sacB*-Kan^R^ cassette or pKan or plasmids derived from pKan in the cyanobacterial cell were grown with the addition of 50 μg/mL Kan. Strains with pSpec or plasmids derived from it were grown with the addition of 30 μg/mL streptomycin (Str) and spectinomcin (Spc). For FFA analysis, mRNA sequence observation, growth rate determination, and measurement of IC_50_, Cyanobacterial strains were also grown in BG-11 liquid media supplied with 10 mM N-[tris(hydroxymethyl) methyl]-2-aminoethanesulfonic acid (TES) NaOH (pH 8.2) in a 15 mL glass test tube 30 °C in an environmental chamber. All chemicals were purchased from Sigma Aldrich (St. Louis, MO), BD (Franklin Lakes, NJ), or Thermo Fisher Scientific (Waltham, MA). The tubes containing cyanobacteria and BG-11 liquid media was incubated with illumination and intermittent shaking for 2–4 d. Once these starter cultures reached an OD_730_ nm of 0.6, the samples were transferred to a 250-mL Erlenmeyer flask with aeration at 100 mL∕ min. This protocol uses TES buffer and air aeration to keep the pH around 8 at the beginning inoculation stages to minimize the lag phase. Once the culture reaches mid-log level phase, the culture was used to test the IC_50_. If instead the culture was used for FFA analysis, 20 mL of BG-11 liquid media without TES was added, as TES negatively affects FFA concentration [8]. This now 30 mL culture was growth to a concentration of 0.8-1 × 10^9^ cells/mL and then subjected to FFA analysis. However, if the culture was used for mRNA analysis, 40 mL of BG-11 was added to the initial 10 mL culture to create a 50 mL culture. Once this reaches the mid-log level phase, 40 mL was removed and analyzed for the presence of specific mRNA sequences. The specific protocols for the measurements of IC_50_, FFA concentrations, and the presence mRNA sequences are explained in detail in subsequent sections.

To determine the growth rate of the strains, an initial OD_730_ of 0.05 was created in a 30 mL 250 mL Erlenmeyer flask. It was placed in the illuminated chamber and aerated at 100 mL/min, but not rotated. The OD_730_ was measured by spectrophotometer every 24 h for 168 h. This was performed in triplicate.

### Transformation of suicide vectors containing the *sacB*-Kan^R^ cassette

To delete the entire gene of interest, initially a suicide vector was designed so that one 700-bp sequence located immediately upstream and one 700-bp sequence located immediately downstream of *sll1080* (flanking regions) were amplified from *Synechocystis* sp. PCC 6803 using a colony PCR and ligated to either end of the *sacB*-Kan^R^ cassette. This now upstream flanking region/*sacB*-Kan^R^ cassette/downstream flanking region nucleotide sequence was digested using restriction enzymes ligated into a restriction enzyme digested pGEM-3Z. The resulting plasmid (pΨ695) was electroporated into *E. coli* Top10 cells and a colony of Top10 cells containing pΨ695 was grown in liquid lysogeny broth (LB) with Miller’s modification [50] at 37 °C overnight with aeration. pΨ695 was then purified from the exponentially grown Top10 cells containing pΨ695 using the Qiagen Plasmid Miniprep Kit (Hilden, Germany) About 100 cyanobacteria cells in 10 μL BG-11 medium were mixed with 400 ng of purified pΨ695 (1–2 μL of DNA dissolved in Elution Buffer (Qiagen)) and incubated for 4 h at room temperature under ambient lighting. Then the mixtures were plated onto a filter membrane (Whatman PC MB 90MM 4 μm (Maidstone, UK)) layered on a BG-11 agar plate. After growth for 24 h on the BG-11 plate at room temperature under ambient lighting, the membrane carrying the cyanobacteria was transferred onto a BG-11 agar plate containing 50 μg∕mL Kan. Once individual colonies appear, they were picked and placed with a sterile loop onto a new BG-11 with 50 μg∕mL Kan agar plate. The cyanobacteria were moved to a BG-11 with Kan plate in order to select for colonies that contain cells that have the *sacB*-Kan^R^ cassette in place of the native *sll0180* gene in the chromosome. This process is defined as homologous recombination in which the suicide vector has homologous regions to the native sequence of the chromosome, allowing the *sacB*-Kan^R^ cassette to be exchanged for *sll0180*. However, in order for *sll0180* to be completely removed from *Synechocystis* sp. PCC 6803 and SD277, the gene must be removed from all copies of the chromosome; this is defined as complete segregation. In order to identify that the *sacB*-Kan^R^ cassette is present in place of the gene of interest in every chromosome copy, a colony PCR was performed using primers that amplify the flanking region both upstream and downstream of *sll0180*, A end sll1080 end F and B end sll0180 end R (Table 2). Regions with the native *sll0180* are 1 kb shorter than regions with the *sacB*-Kan^R^ cassette when observed on 0.8% agarose DNA gel electrophoresis. If the native gene persisted, then the colony was picked using a sterile loop and plated on a new BG-11 plate containing 50 μg∕mL Kan. Again, after growth for 2–3 days in an illuminated chamber at 30 °C a colony of cyanobacteria was suspended in 3 μl of dH_2_O and a PCR was performed using the primers for the two flanking regions. These steps were repeated until the native gene sequence was not observed, leaving the cyanobacteria with only the *sacB*-Kan^R^ cassette in place of *sll0180*. The same protocol was performed to replace *slr2131* in SD100 and SD277. The one exception is which primers were used to identify whether or not *slr2131* was present in the cell since *slr2131* has almost the identical nucleotide sequence length as the *sacB*-Kan^R^ sequence. Because of the similar sequence lengths, a new set of primers was created to amplify a 373 bp region both located with *slr2131* (2131 w/in A and 2131 w/in B) (Table 2) were used to identify whether or not *slr2131* was present in the cell. And finally, to ensure that the 3.2 kb nucleotide sequence of *sacB*-Kan^R^ was present between the flanking regions of *slr2131*, the primers encoding the *slr2131* flanking region sequences were used. The DNA electrophoresis gels depicting the deletion confirmation are in Additional file 1: Figure S3.

### Conjugation to add an expression vector containing an *E. coli* gene

Two expression vectors were created for the currently presented research based on derivatives of RSF1010 by the authors, with each conferring resistance to either Kan or Spc and Str, named pKan and pSpec, respectively. The *aphA* gene from *E. coli* MG1655 is expressed in pKan, while the *aadA* gene from the *E. coli* R100 plasmid is expressed in pSpec. Each expression vector contains the P_trc_ promoter that was used constitutively to express the *E. coli* gene, either *acrA* or *acrB*. *E. coli acrA* or *acrB* genes were amplified from the *E. coli* chromosome using isolated genomic DNA (Wizard Genomic DNA Purification Kit, Promega, Madison, WI) using the following primers: 5’ AcrA F, 3’ AcrA R, 5’ AcrB F, 3’ AcrB R (Table 2). *E. coli acrA* or *acrB* genes were individually ligated into the multiple cloning site adjacent to P_trc_ of pKan and pSpec respectively, resulting in vectors named p*acrA* and p*acrB* (Table 1). Additionally, *acrA* was ligated into pSpec resulting in pS*acrA*. In order to move these expression vectors into SD277 or SD100, conjugation must be performed to alleviate the effects of native restriction enzyme digestion in the cyanobacterial cells using an established method [51, 52]. To begin the process of conjugation, two cultures of Top10 *E. coli* cells containing either pRL443, which mobilizes the conjugational pair, facilitating the transfer of plasmids between cells, or pRL528 (a helper plasmid) and the expression vector (pKan, pSpec, p*acrA*, or p*acrB*) were grown in 15 mL of liquid lysogeny broth (LB) using the Miller modification [50] overnight at 37 °C. Simultaneously, 10 mL of the cyanobacteria strain was grown in a liquid BG-11 culture to an OD_730_ of 0.6. Then, all three strains, both *E. coli* and cyanobacteria, underwent a washing process in which the initial sample was centrifuged for 10 min at 3000 x g. The supernatant was removed, and the sample pellet was resuspended in 10 mL of sterilized BG-11. This process was repeated two more times. After the final wash, the strains were resuspended in 5 mL of BG-11. The process of conjugation was begun by the addition of 1 mL of the cyanobacterial strain to 2 mL of the *E. coli* strain with pRL443 and 2 mL of the *E. coli* strain containing both the expression vector and pRL528. These cultures then sat for 20 min at room temperature. Then 400 μL of each of the conjugation and control groups were spread onto a nitrocellulose membrane that had been placed on BG-11 with 0.5% LB media. This sat on the lab bench overnight. The next morning the plates were put in an illuminated chamber (SANYO) at 30 °C until a light green culture of bacteria appeared on the surface of the filter (usually after a day or two). The nitrocellulose membrane was moved to BG-11 with antibiotics corresponding to the expression vector to select for cyanobacterial strains containing the desired expression vector. Individual, bright green colonies grew on the nitrocellulose membrane within a week that potentially represented successfully modified cyanobacterial cells. The presence of the *E. coli* gene was confirmed using colony PCR using the same primers that were initially used to amplify the corresponding nucleotide sequence from the *E. coli* chromosome as described in the following section.

### Confirmation of gene replacement or addition using colony PCR

The colony PCR is performed by suspending the cyanobacterial cells from the colony of interest into 3 μL of dH_2_O in a 200 μL-PCR tube. The suspension then proceeds through a triplicated freeze/thaw cycle from -80 °C to 55 °C. Then GoTaq DNA polymerase (Promega, Madison, WI) is used in a reaction mixture with the primers explained in previous sections to amplify the targeted DNA sequence. The thermocycler temperature and time conditions were as follows: Initial denaturation for 2 min at 95oC, then the next 3 conditions were repeated 30 times of denaturation for 1 min at 95oC, annealing for 1 min at 56oC, and extension for 1.5 min at 72oC, and finally, the final extension step occurs for 7 min at 72oC.

### FFA analysis

The cyanobacterial strain was grown to a concentration of 0.8-1 × 10^9^ cells/mL and 20 mL of the culture was removed and centrifuged at 6000 x g. The supernatant was placed into a new centrifuge tube after being filtered for any cells that remained suspended in the liquid media. The cell pellet was then processed for intracellular FFA concentration analysis using a modified Folch method as described previously [53]. Briefly, 3 mL of a ratio of 2:1 of chloroform:methanol mixture was added to the pellet, which is vortexed at room temperature for 24 h at low speed. The solvent and resuspended pellet mixture is filtered using a PTFE membrane resulting in the solvent with FFAs, which was then evaporated using positive nitrogen evaporator (N-EVAP 111, Organomation, Berlin, MA) until the sample was dry. Both the dried sample from the extracted pellet and the collected supernatant from the initial centrifugation were processed for FFA concentrations by adding 10 mL of hexane and 200 μL of 3 M H_3_PO_4_. These samples were agitated on a rotator at 180 rpms for 30 min at 37 °C and then the sample was centrifuged at 6000 x g for 5 min. 5 mL of the resulting uppermost layer (hexane layer) was removed and moved to a glass tube (13 × 100 mm) and dried on a nitrogen evaporator. The dried samples were then dissolved in 1 mL of hexane and analyzed by a gas chromatograph (GC) (Shimadzu GC 2010) with a Supelco Nukol capillary column (30 m × 0.53 mm × 0.5 μm) and flame ionization detector [54]. GC operating conditions were as follows: split ratio 1∶5; inject volume 1 μL; N_2_ carrier gas with a constant flow rate 30 mL∕ min; H_2_ 40 mL∕ min, air 400 mL∕ min, make up gas (nitrogen) 5 mL∕ min; injector and detector temperature 250 °C; and oven temperature was started at 100 °C and increased at a rate of 10 °C∕ min to 220 °C and held for 10 min. Each FFA compound was identified by comparing its retention time with that of a standard FFA (Sigma Aldrich, St. Louis, MO, USA). The GC was used to test for the presence of the following hydrocarbons: lauric acid, myristic acid, palmitic acid, stearic acid, pentadecanoic acid, margaric acid, 6-[(10Z)-heptadecenyl] salicylic acid, hydroxyhexadecanoic acid, and hydroxytetradecanoic acid. Only lauric, myristic, palmitic, and stearic acids were observed in consistent concentrations greater than 1 mg/mL. FFA concentrations in samples were quantified based on the area under the chromatogram peak in comparison with the standards. Each sample was run through the gas chromatograph in triplicate and the concentration of each FFA present is averaged with the three data points. Each strain underwent this entire growth, supernatant/pellet separation, and analysis process in triplicate.

### Half maximal inhibitory concentration (IC_50_) determination

Once the cyanobacterial strains reached mid-level growth phase as described in a previous section, they were assessed for the IC_50_ to Amp, Cm, and Ery. A 96-well flat bottom plate (Corning Inc., Corning, NY) in which each well was filled with a 50 μL solution containing a range of concentrations of each antibiotic (Amp, Cm, or Ery) that created final concentrations between 10 ng/mL to 10 mg/mL was used to inoculate 50 μL of the cyanobacterial sample to create a final volume of 100 μL. Each cyanobacterial sample in the well of the plate was measured at an initial OD_790_ between 0.35 and 0.4 using a microplate reader (Biotek Synergy H1, Winooski, VT). Each 96-well plate was placed in an illuminated chamber at 30 °C on a rotator (60 rpm) for 72 h at which point the final OD_790_ was measured of each sample.

### RT-PCR analysis of mRNA

A 40 mL culture of a cyanobacterial strain is grown to mid-log level phase as explained previously, which was then centrifuged at 6000 x g for 10 min to pellet the cells. The supernatant was removed, and the remaining pelleted cells were resuspended in 500 μL of BG-11 media and 1 mL of RNAprotect Cell Reagent (Qiagen). Each sample was stored at -20 °C until all samples were ready for RNA isolation. For RNA extraction, the Qiagen RNeasy Kit was used. Briefly, the stored samples were thawed and pelleted using a centrifuge at a force of 6000 x g for 10 min. The supernatant was removed, and the pellet was resuspended in 700 μL of RLT Buffer (Qiagen) and transferred to a 2 mL locking lid microcentrifuge tube (Eppendorf, Hamburg, Germany) containing 0.2 g of 0.25 mm-diameter acid washed glass beads (Sartorius Stedim, Göttingen, Germany). The mixture was placed in a Bullet Blender Storm 24 homogenizer (Next Advance, Troy, NY) and homogenized for 5 min at the maximum speed. The remaining extraction steps were followed exactly as Qiagen recommended using the RNeasy Protect Bacterial Mini Kit. The mRNA concentration of each sample was quantified using a NanoDrop 2000c spectrophotometer (Thermo Fisher Scientific, Waltham, MA). Each mRNA sample (1 μg) was treated with one unit of RQ1 RNase-free DNase (Promega) according to the manufacturer’s instructions. To create the cDNA, 250 ng of RNA was transcribed using the Qiagen One-Step RT-PCR Kit in a final reaction volume of 50 μL and then placed in the thermal cycler using the manufacturer’s recommendations, using the primers previously published to identify mRNA expression of *E. coli* genes *acrA* and *acrB* [55] (Table 2). Separately, a set of primers from previously published work was used to identify *petB* mRNA (Table 2), a constitutively expressed *Synechocystis* sp. PCC 6803 gene [56], ensuring that mRNA was isolated from every cyanobacterial strain, even in those that did not possess any *E. coli* genes.

To ensure there was no DNA contamination in the mRNA samples after the DNase treatment, each mRNA sample underwent a conventional PCR using GoTaq polymerase, appropriate reagents, and the corresponding primers to the gene of interest (*acrA* or *acrB*).

### Statistics

Statistical analyses were performed using the GraphPad Prism 5 software package (GraphPad Software, San Diego, CA). A non-linear regression curve using the log (chemical concentration) versus the cell concentration in 4-parameter variable slope model was used to identify the IC_50_ as well as the 95% confidence intervals of that concentration. To compare the total FFA concentration of strains, a Mann-Whitney test was used. To determine the difference of growth rate of strains, a one-way ANOVA was used in which each time point was considered a matched result to be compared. Differences were considered significant at a *P* value of < 0.05. *p < 0.05, **p < 0.005, ***p < 0.0005, **** < 0.0001.

## Additional file

Additional file 1: **Figure S1.** Electrophoresis Gel displaying the DNA fragments created from RT-PCR of (A) *E. coli* genes *acrA* and *acrB* and (B) of the *Synechocystis* sp. PCC6803 *petB* gene. **Figure S2.** Optical density measurements at 730 nm wavelength every 24 h for 168 h of (A) SD277 and its mutant and complementation derivatives, (B) SD100 and its mutant and complementation derivatives, (C) SD277 and its plasmid addition derivatives, and (D) SD100 and its plasmid addition derivatives. The culture conditions were as follows: illumination was 50 μmol photons m^− 2^ s^− 1^, temperature was 30 °C, aeration of filtered air was pumped at a rate of 100 mL/min, and CO^2^ concentration was the normal atmospheric concentration. **Figure S3.** Electrophoresis gel displaying the DNA fragments created from PCR of using primers of either end fragments of *sll0180* in (A) SD100, SD100 ∆*sll0180*, SD100 ∆*sll0180* pS*acrA* and (B) SD277, SD277 ∆*sll0180*, SD277 ∆*sll0180* pS*acrA.* Electrophoresis gel displaying the DNA fragments created from PCR using primers of either end fragments of *slr2131* in (C) SD100, SD100 ∆*slr2131*, SD100 ∆*slr2131* p*acrB*, SD277, SD277 ∆*slr2131*, and SD277 ∆*slr2131* p*acrB*. Electrophoresis gel displaying the DNA fragments created from PCR of using primers of a region within *slr2131* in (D) SD100, SD100 ∆*slr2131*, SD100 ∆*slr2131* p*acrB*, SD277, SD277 ∆*slr2131*, and SD277 ∆*slr2131* p*acrB*. (DOCX 6501 kb)

## Abbreviations

ABC: ATP-binding cassette
Amp: Ampicillin
Cm: Chloramphenicol
FFA: Free fatty acid
GC: Gas chromatograph
IC_50_: Half maximal inhibitory concentration
Kan: Kanamycin
LB: Lysogeny Broth
MFP: Membrane fusion protein
OD: Optical Density
PCR: Polymerase Chain Reaction
RT-PCR: Reverse transcriptase-polymerase chain reaction
SDS: Sodium dodecyl sulfate
Spc: Spectinomcin
Str: Streptomycin
TES: N-[tris(hydroxymethyl) methyl]-2-aminoethanesulfonic acid
