# Correction to: characterizing active transportation mechanisms for free fatty acids and antibiotics in *Synechocystis* sp. PCC 6803

**DOI:** 10.1186/s12896-019-0505-y

**Published:** 2019-02-13

**Authors:** Matthew P. A. Bellefleur, Soo-Young Wanda, Roy Curtiss III

**Affiliations:** 1 School of Life Sciences, Arizona State University, 427 E. Tyler Mall, Tempe, AZ 85287 USA; 2 College of Veterinary Medicine, University of Florida, 2015 SW 16th Ave, Gainesville, FL 32608 USA; 3 Faculty of Human Sciences, Waseda University, 1-104 Totsukamachi, Shinjuku-ku, Tokyo, 169-8050 Japan

**Correction to: Bellefleur et al. BMC Biotechnology (2019) 19:5**

**https://doi.org/10.1186/s12896-019-0500-3**

Following publication of the original article [1], the author reported that the gene/protein names of slr2131 and sll0180 were swapped in the Discussion section. The details of the correction are mentioned below:

Excerpt with swapped protein/gene names in the original article:

Previous research performed by Gonçalves et al. (2018) supports the hypothesis that Sll0180 is necessary for native efflux in that the removal of sll0180 from Synechocystis sp. PCC 6803 caused a significant inhibition of growth due to the presence of Cm [30]. However, Gonçalves et al. (2018) did not observe a significant inhibition of growth associated with the removal of slr2131 from Synechocystis sp. PCC 6803 due to the presence of Cm, as shown in the currently presented research.

Excerpt with correct protein/gene names:

Previous research performed by Gonçalves et al. (2018) supports the hypothesis that Slr2131 is necessary for native efflux in that the removal of slr2131 from Synechocystis sp. PCC 6803 caused a significant inhibition of growth due to the presence of Cm [30]. However, Gonçalves et al. (2018) did not observe a significant inhibition of growth associated with the removal of sll0180 from Synechocystis sp. PCC 6803 due to the presence of Cm, as shown in the currently presented research.

## References

[1] MPA B (2019). Characterizing active transportation mechanisms for free fatty acids and antibiotics in *Synechocystis* sp. PCC 6803. BMC Biotechnol.

