# Supplementary material for: Characterizing active transportation mechanisms for free fatty acids and antibiotics in Synechocystis sp. PCC 6803
Source: BMC Biotechnol. 2019 Jan 10;19:5. doi: 10.1186/s12896-019-0500-3 (PMC6329066; doi:10.1186/s12896-019-0500-3)
Supplement: Supplementary file 1 — Figure S1. Electrophoresis Gel displaying the DNA fragments created from RT-PCR of (A) E. coli genes acrA and acrB and (B) of the Synechocystis sp. PCC6803 petB gene. Figure S2. Optical density measurements at 730 nm wavelength every 24 h for 168 h of (A) SD277 and its mutant and complementation derivatives, (B) SD100 and its mutant and complementation derivatives, (C) SD277 and its plasmid addition derivatives, and (D) SD100 and its plasmid addition derivatives. The culture conditions were as follows: illumination was 50 μmol photons m− 2 s− 1, temperature was 30 °C, aeration of filtered air was pumped at a rate of 100 mL/min, and CO2 concentration was the normal atmospheric concentration. Figure S3. Electrophoresis gel displaying the DNA fragments created from PCR of using primers of either end fragments of sll0180 in (A) SD100, SD100 ∆sll0180, SD100 ∆sll0180 pSacrA and (B) SD277, SD277 ∆sll0180, SD277 ∆sll0180 pSacrA. Electrophoresis gel displaying the DNA fragments created from PCR using primers of either end fragments of slr2131 in (C) SD100, SD100 ∆slr2131, SD100 ∆slr2131 pacrB, SD277, SD277 ∆slr2131, and SD277 ∆slr2131 pacrB. Electrophoresis gel displaying the DNA fragments created from PCR of using primers of a region within slr2131 in (D) SD100, SD100 ∆slr2131, SD100 ∆slr2131 pacrB, SD277, SD277 ∆slr2131, and SD277 ∆slr2131 pacrB. (DOCX 6501 kb) [file 12896_2019_500_MOESM1_ESM.docx]

Additional file 1

Characterizing active transportation mechanisms for free fatty acids and antibiotics in *Synechocystis* sp. PCC 6803 using genome editing and plasmid-encoded *Escherichia coli* genes

Matthew P.A. Bellefleur^1,2^, Soo-Young Wanda^1,2^, Roy Curtiss, III ^1,2^

^1^Arizona State University, School of Life Sciences, 427 E. Tyler Mall, Tempe, AZ, USA 85287

^2^University of Florida, College of Veterinary Medicine, 2015 SW 16^th^ Ave, Gainesville, FL, USA 32608

*correspondence to mbellefl@asu.edu

**Figure S1 S2**

**Figure S2 S3**

**Figure S3 S4**

**S1**

**Figure S1**

Electrophoresis Gel displaying the DNA fragments created from RT-PCR of (A) *E. coli* genes *acrA* and *acrB* and (B) of the *Synechocystis* sp. PCC6803 *petB* gene.

**S2**

**Figure S2**

Optical density measurements at 730 nm wavelength every 24 h for 168 h of (A) SD277 and its mutant and complementation derivatives, (B) SD100 and its mutant and complementation derivatives, (C) SD277 and its plasmid addition derivatives, and (D) SD100 and its plasmid addition derivatives. The culture conditions were as follows: illumination was 50 μmol photons m^-2^ s^-1^, temperature was 30^o^C, aeration of filtered air was pumped at a rate of 100 mL/min, and CO^2^ concentration was the normal atmospheric concentration.

**S3**

**Figure S3**

Electrophoresis gel displaying the DNA fragments created from PCR of using primers of either end fragments of *sll0180* in (A) SD100, SD100 ∆*sll0180*, SD100 ∆*sll0180* pS*acrA* and (B) SD277, SD277 ∆*sll0180*, SD277 ∆*sll0180* pS*acrA.* Electrophoresis gel displaying the DNA fragments created from PCR using primers of either end fragments of *slr2131* in (C) SD100, SD100 ∆*slr2131*, SD100 ∆*slr2131* p*acrB*, SD277, SD277 ∆*slr2131*, and SD277 ∆*slr2131* p*acrB*. Electrophoresis gel displaying the DNA fragments created from PCR of using primers of a region within *slr2131* in (D) SD100, SD100 ∆*slr2131*, SD100 ∆*slr2131* p*acrB*, SD277, SD277 ∆*slr2131*, and SD277 ∆*slr2131* p*acrB*.

**S4**
